# Atopic Dermatitis in the Era of Exposome and Artificial Intelligence

**DOI:** 10.3390/ijms27209209

**Published:** 2026-10-16

**Authors:** Sara Manti, Federica Li Pomi, Luca Gammeri, Francesco Borgia, Giuseppe Murdaca, Sebastiano Gangemi

**Affiliations:** 1 Pediatric Unit, Department of Human Pathology in Adult and Developmental Age “Gaetano Barresi”, University of Messina, 98125 Messina, Italy; sara.manti@unime.it; 2 Department of Precision Medicine in Medical, Surgical and Critical Care (Me.Pre.C.C.), University of Palermo, 90127 Palermo, Italy; federicalipomi@hotmail.it; 3 Department of Biomedical and Dental Science and Morphofunctional Imaging, University of Messina, 98125 Messina, Italy; luca.gamm1993@gmail.com; 4 Section of Dermatology, Department of Clinical and Experimental Medicine, University of Messina, 98125 Messina, Italy; fborgia@unime.it; 5 Department of Internal Medicine, University of Genova, 16132 Genova, Italy; 6 Allergology and Clinical Immunology Unit, San Bartolomeo Hospital, 19038 Sarzana, Italy; 7 Allergy and Clinical Immunology Unit, Department of Clinical and Experimental Medicine, University of Messina, 98125 Messina, Italy; sebastiano.gangemi@unime.it

**Keywords:** atopic dermatitis, eczema, exposome, artificial intelligence, machine learning, precision medicine, air pollution, microbiome, biomarkers, digital health, flare prediction

## Abstract

Atopic dermatitis (AD) is a heterogeneous, relapsing inflammatory skin disease in which epidermal barrier dysfunction, immune dysregulation, microbial imbalance, genetics, and the environment interact continuously over time. Although a substantial body of literature has linked AD with air pollution, weather, allergens, stress, infections, sleep disturbance, skin care practices and microbial perturbation, most studies have assessed single exposures in isolation. This reductionist approach only partially reflects the real-world biology of AD, where patients experience dynamic combinations of external and internal exposures whose effects are non-linear, cumulative and modified by age, phenotype, treatment and susceptibility. The exposome paradigm offers a broader conceptual lens by considering the totality of exposures over the life course. However, exposomic research in AD is methodologically challenging because the data are multimodal, high-dimensional, time-dependent, and often incomplete. Artificial intelligence (AI) can help address this complexity by integrating heterogeneous data layers, modelling temporal dependencies, discovering latent patterns, and generating individualized predictions. This narrative review examines current evidence and proposes a theoretical framework that integrates AI and the exposome in AD. We define the principal domains of the AD exposome, describe a multilevel analytical architecture spanning environmental, clinical, behavioural, microbiome, and multi-omics data. We distinguish applications evaluated in AD studies from exploratory analyses and proposed objectives: prediction of annual flare burden remains exploratory, whereas exposome-informed short-term flare alerts are proposed applications. Although selected AI applications have been evaluated in AD, the added predictive value of exposure data and the clinical utility of a fully integrated exposome–AI system remain to be established. We also outline key methodological and translational priorities, including exposure assessment, temporality, treatment-confounding, interpretability, fairness, data governance, and external validation. The proposed framework offers a structured basis for future research, with clinical translation dependent on prospective validation and evidence of benefit to patient care.

## 1. Introduction

Atopic dermatitis (AD) is among the most common chronic inflammatory skin diseases worldwide. It represents a major cause of itch, sleep disruption, psychosocial burden, and chronic health care use across the life course [1,2]. Its clinical expression is heterogeneous, with substantial interindividual variation in age of onset, anatomical distribution, inflammatory profile, comorbidities, severity profile, persistence into adulthood, and response to both topical and systemic treatments [2,3,4,5,6]. This heterogeneity is increasingly understood as the consequence of dynamic, bidirectional interactions among epidermal barrier dysfunction, type 2-skewed and non-type 2 immune responses, cutaneous microbial dysbiosis, genetic and epigenetic susceptibility, and a wide range of environmental modifiers [2,3,4,5,6].

Specifically, an expanding body of evidence supports the relevance of the exposome in AD, encompassing external exposures and internal biological processes that may influence disease risk and expression across the life course [7,8,9,10,11,12,13,14,15,16]. Associations have been described for a broad range of environmental and behavioural exposures, including outdoor and indoor air pollutants, meteorological variability, ambient temperature and humidity, urbanization, tobacco smoke, psychosocial stress, sleep disruption, infections, topical and household products, as well as alterations in the skin and gut microbiota. These exposures may interact, converging on key pathogenic mechanisms of the disease. Their clinical impact may vary not only with age and individual susceptibility, but also with factors such as disease phenotype or the chronicity of exposure [7,8,9,10,11,12,13,14,15,16].

Despite this, these factors are often studied one at a time. Such studies may not fully capture combinations of exposures, cumulative burden, or treatment confounders [17,18]. In the clinical setting, however, patients are not exposed to a single trigger but experience overlapping constellations of environmental, behavioural, psychosocial, and biological influences. These constellations vary across hours, days, seasons, and developmental stages.

The exposome concept is particularly interesting when applied to AD because it explicitly frames disease biology in relation to cumulative exposures across the lifespan and incorporates external general, external specific, and internal domains [7,8,9]. However, the statistical and biological complexity of these data poses significant analytical challenges. Variables are often multiple, interdependent, and nonlinear. In some cases, variables are partially missing, and distinct exposure levels operate on different timescales and may interact or modify one another. Such complexity closely matches the type of analytical setting in which artificial intelligence (AI) may offer particular value [19,20,21]. Machine learning (ML) and related computational approaches can model nonlinear interactions and handle multimodal data.

Furthermore, they can identify patient subgroups and estimate individualized risk. The use of these AI approaches could support decision-making in complex chronic diseases such as AD [22,23,24]. In this context, exposomes and AI are not simply adjacent fields but complementary methods. By studying the exposome, it is possible to capture the dynamics of environmental influences.

The rationale for integrating AI and exposome in AD is therefore both conceptual and practical. Conceptually, AD is a paradigmatic disease in which host susceptibility and a dynamic environmental context interact repeatedly over time [22,23,24,25]. Practically, recent works have shown that AD severity can be predicted from longitudinal data or assessed from clinical images [26,27,28]. Specifically, artificial neural network (ANN) analysis has already been applied to the relationship between AD severity, air pollutants, and weather changes, supporting the feasibility of environmental AI modelling in this field [27].

Building on these premises, this paper presents a narrative review and proposes a theoretical framework for integrating AI and the exposome in AD. We aim to define the major domains of the AD exposome, describe the architecture of an AI-enabled exposomic pipeline, and identify potential clinically relevant outputs such as severity prediction, flare forecasting, and exposome-sensitive patient subgroups. Finally, we aim to summarize methodological priorities and translational challenges for future research.

## 2. Methods

This narrative review was informed by literature searches in PubMed/MEDLINE, Scopus, and Google Scholar, covering publications available up to 1 May 2026. Search terms included combinations of “atopic dermatitis” with “exposome”, “environmental exposure”, “artificial intelligence”, “machine learning”, “deep learning”, and “prediction”. Articles were selected for their relevance to the biological domains of the AD exposome, existing AI applications in AD, and methodological considerations for the proposed framework. Original studies were used to discuss specific applications, while reviews and methodological publications provided biological and analytical context. The literature was synthesized qualitatively to distinguish findings demonstrated in AD studies from exploratory applications and proposed research directions. No systematic evidence grading or meta-analysis was undertaken.

## 3. Why an Exposome–AI Perspective Is Needed in AD

A major limitation of the current literature is the persistence of a single-exposure paradigm, whereby environmental and biological factors are often examined in isolation. Air pollutants are assessed independently of meteorological variables; humidity is considered separately from temperature; psychological stress is analysed without accounting for sleep disturbances; and microbial findings are often interpreted without sufficient consideration of their environmental context [7,8,9,10,11,14,15,16,29,30]. While such reductionist approaches facilitate statistical interpretation and yield clear associations, they may not adequately reflect the complex biological reality of co-exposure. In real-world settings, individuals are simultaneously subjected to multiple interacting factors. For example, in the same patient, multiple factors may simultaneously contribute to a single disease flare (high exposure to particulate matter, sudden temperature fluctuations, increased nocturnal itching, increased psychological stress, inconsistent adherence to topical therapies, and excessive overgrowth of Staphylococcus aureus) [7,8,9,10,11,14,15,16,29]. These factors represent different dimensions: environmental exposures, symptoms, behavioural factors, and microbial changes should not be treated as equivalent exposures or assumed to precede disease worsening. These factors may interact in synergistic, antagonistic, or threshold-dependent ways that conventional linear or single-variable models may not fully capture.

Temporality represents another major limitation; indeed, environmental and behavioral exposures act on different time scales. Some factors, such as fluctuations in air pollution or temperature, may exert acute effects over short periods, such as hours or days. In contrast, other exposures, including urbanization and economic disadvantage, accumulate gradually over prolonged periods [7,8,9,10,11,14,15,16,31]. Susceptibility to these exposures may vary across life stages. Environmental factors may differentially influence disease onset and progression across age groups. In parallel, disease trajectories appear heterogeneous and show variable patterns of persistence, remission, and exacerbation [32,33,34,35,36,37]. Consequently, cross-sectional studies and clinical investigations with limited longitudinal follow-up may incompletely capture the intrinsically dynamic and evolving nature of the disease.

A third key issue is variation in susceptibility across individuals. Exposure–response relationships are not uniform but may vary substantially depending on host-specific factors. These include epidermal barrier integrity, filaggrin mutation status, underlying inflammatory endotypes, microbiome composition, as well as demographic and psychosocial variables such as ethnicity, age, sex, and psychological burden. Treatment intensity and adherence are additional, potentially time-varying contextual factors rather than intrinsic susceptibility traits [5,6,18,30,31,32,34,36,38,39,40]. It is now well established that AD is not a single, homogeneous disease entity, but a spectrum of related conditions that share common clinical manifestations while being underpinned by partially distinct molecular mechanisms [41,42,43]. In such a heterogeneous context, reliance on average effects derived from mixed populations may obscure clinically meaningful subgroups and mask differential responses to environmental exposures or therapeutic interventions.

Taken together, these limitations underscore the need for a more comprehensive and integrative framework. Rather than modelling environmental exposures, host biology, temporal dynamics, and treatment factors in isolation or in a stepwise manner, future research should aim to consider these dimensions jointly. The concept of the exposome offers a valuable overarching framework for capturing the totality of environmental exposures across the lifespan. At the same time, advances in AI provide analytical tools for handling high-dimensional, complex, and interacting data structures. Whether their joint analysis improves prediction or clinical decision-making in AD remains to be demonstrated.

## 4. The Atopic Dermatitis Exposome: Domains and Biological Meaning

The AD exposome can be conceptualized as three interrelated domains: the external general exposome, the external specific exposome, and the internal exposome. These domains are not discrete compartments, but dynamically interacting layers that collectively shape disease susceptibility, phenotypic expression, and longitudinal course [7,8,9]. The external general exposome comprises broad contextual and macroenvironmental influences, including outdoor air pollution, meteorological conditions, ultraviolet radiation (UV), traffic-related exposures, urban form, green space, climate, environmental tobacco smoke, housing characteristics, and socioeconomic conditions [7]. These exposures may affect AD through convergent mechanisms involving oxidative stress (OS), disruption of epidermal barrier function, neuroimmune activation, and perturbation of microbial ecology. Among them, air pollution has been extensively investigated in both epidemiological and mechanistic studies, with particulate matter, nitrogen dioxide, ozone, and traffic-related pollutant mixtures repeatedly associated with disease onset, exacerbation, and health care utilization [10,11,12,13]. Relevant factors include climatic factors, which can modulate fundamental pathogenic elements like transepidermal water loss (TEWL), skin integrity, and inflammatory activity [14,15,16]. Therefore, the general external exposome constitutes the ecological background against which individual vulnerability is expressed.

The external specific exposome refers to more individual-level exposures encountered directly in everyday life, including allergens, detergents, cosmetics, emollient use, bathing practices, textiles, diet, infections, antibiotics, psychosocial stress, sleep disturbance, and physical activity [18,44,45,46]. These exposures are especially important from a translational perspective because many are modifiable and potentially amenable to high-resolution, real-world monitoring through digital tools, wearable technologies, and repeated patient-reported measures. These factors may act not only as triggers of acute worsening but also as determinants of chronic disease persistence. Psychosocial stress and sleep disturbances can play a bidirectional role in the pathogenesis of AD. Indeed, both factors can arise from itching and inflammation, but they can also exacerbate them, reinforcing a self-perpetuating disease cycle [18,45].

The internal exposome encompasses endogenous biological processes and responses that may reflect interactions between exposure history and host susceptibility. It encompasses OS pathways, neuroendocrine and hormonal signalling, lipid abnormalities, microbiome alterations, inflammatory mediators, circulating and tissue biomarkers, and broader multi-omics layers [7,34]. In AD, this domain is particularly informative since it may capture the downstream biological consequences of repeated interactions between environmental inputs and a vulnerable epidermal–immune axis [47]. Filaggrin-related biology is also highly relevant in this context, although it lies at the intersection of the internal exposome, host susceptibility, and core disease pathobiology [48]. Filaggrin mutation status, however, represents a baseline susceptibility factor rather than an exposure-induced biological response. These internal readouts may help explain why patients with apparently similar clinical phenotypes differ substantially in persistence, flare propensity, treatment response, and comorbidity burden.

Although the internal exposome overlaps with AD pathobiology, baseline susceptibility, external exposures, internal biological responses, and clinical disease activity should be distinguished analytically. Internal measurements may reflect exposure-related responses, intrinsic disease processes or treatment effects; they should therefore not automatically be interpreted as consequences of environmental exposure.

The principal contribution of the exposome concept to AD is thus not merely classificatory. Rather, it shifts the field away from reductionist models centred on isolated triggers and toward a systems-level perspective in which environmental context, host response, and intrinsic susceptibility interact across the life course. Framed in this way, the exposome is not simply a taxonomy of risk factors, but a biologically meaningful scaffold for understanding disease heterogeneity and for investigating more precise predictive, preventive, and therapeutic strategies.

The exposome framework generates data structures that challenge traditional biostatistical methods. These datasets have distinct characteristics since they are high-dimensional, heterogeneous, and complex, and they typically involve repeated, irregularly sampled measurements, with spatial dependencies and multimodal inputs ranging from environmental sensors to clinical and biological data. Variables are often highly correlated and reflect real-world co-exposure. Conversely, missing data are frequent and not always random. Furthermore, interactions between exposures are expected [27,49,50,51].

ML methods for structured data (penalized regression, random forests, gradient boosting, and Bayesian approaches) can support prediction from clinical and exposure variables, with variable selection or predictor ranking depending on the method used. Furthermore, tree-based methods can model nonlinear relationships and interactions, whereas standard linear or logistic regression requires appropriate nonlinear or interaction terms to represent these relationships. These techniques may support individualized risk prediction beyond population averages [52,53]. For longitudinal prediction, autoregressive and Bayesian state-space models can account for previous disease activity and temporal dependencies, as demonstrated in AD severity modelling.

Second, deep learning methods offer approaches to feature extraction and data integration. Neural networks can process images, wearable sensor outputs, longitudinal symptom records, and high-dimensional omics data. Convolutional neural networks have been evaluated for image-based assessment of AD signs, while multimodal architectures may combine information from different data sources when sufficient, appropriately aligned training data are available [28,54]. This capacity for multimodal integration may be relevant in AD, where disease expression reflects complex interactions between environmental, biological, and behavioural factors [51,52,53].

Furthermore, unsupervised learning approaches can identify data-driven patient clusters without resorting to predefined categories. By uncovering hidden structures in data, these methods may support more refined stratification of heterogeneous patient populations and contribute to precision medicine [39,51,52].

Explainable AI (XAI) tools can help clarify which variables contribute to a model’s predictions and how their contribution varies over time. These approaches make model outputs more transparent, but do not establish whether the identified predictors are causes of disease worsening or explain the underlying biological mechanisms [55]. Causal inference addresses a separate question: whether modifying an exposure or treatment would change a clinical outcome. In AD, this distinction is particularly important because worsening disease may lead to treatment intensification, which in turn affects subsequent disease activity. Investigating causal effects therefore requires an appropriate study design and explicit consideration of treatment-related confounding; predictive accuracy alone is insufficient [56].

AI applications in AD are already emerging, including image-based severity scoring systems, automated SCORAD pipelines, models for daily severity prediction, microbiome–transcriptome classifiers, and wearable devices for monitoring nocturnal scratching. More recent tools also address prediction of annual flare burden and severity stratification [56,57]. However, these applications demonstrate the feasibility of specific tasks, rather than the clinical utility of a fully integrated exposome–AI system. Future progress will require testing whether adding environmental and biological data improves prediction beyond clinical information and previous disease activity, and whether using these predictions to guide care improves patient outcomes.

## 5. Proposed Theoretical Framework

**Figure 1:**
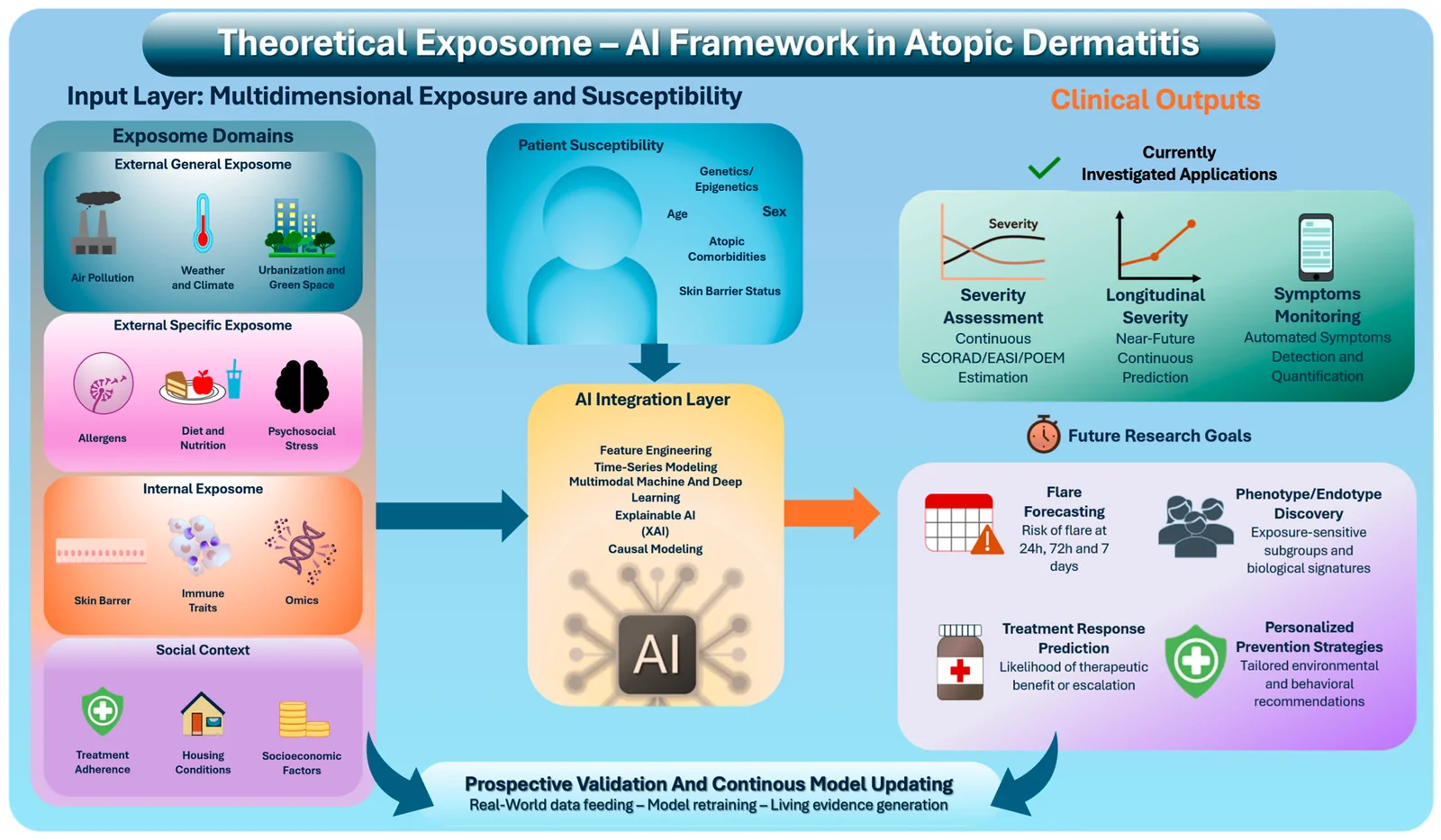
Theoretical framework based on the exposome and AI for atopic dermatitis. The figure summarizes the integration of external and internal exposome domains, patient susceptibility factors, and AI-based analytical levels to generate clinically relevant outcomes. Current applications involve assessing disease severity using validated scores and the longitudinal monitoring of severity and symptoms. Potential future applications aim to predict flare-ups, identify phenotypes and endotypes, and stratify treatment response to develop personalized prevention strategies.

We propose an AI-enabled exposome framework for AD, structured into six interconnected layers. Each layer addresses a key dimension of disease complexity, and together they are intended to support a more integrated and dynamic understanding of disease patterns and their potential implications for management (Supplementary Table S1, Figure 1).

### 5.1. Input Layer: Multidimensional Exposure Capture

This first layer focuses on comprehensive data acquisition across multiple domains. Relevant inputs include clinical assessments, patient-reported outcomes, and detailed treatment records, combined with geospatial linkage to environmental databases capturing air pollution and meteorological variables. Additional sources encompass indoor monitoring systems, wearable sensors, digital symptom diaries, microbiome profiling, and both targeted and untargeted omics platforms [9,11,12,15,16,31,46,48,56]. The system should collect standardized measures of disease severity (e.g., EASI, SCORAD, POEM), flare occurrence, pruritus intensity, sleep quality, treatment exposure and adherence, as well as a core set of environmental variables. The aim is to construct a high-resolution, real-world representation of the patient’s exposome.

### 5.2. Temporal Layer

Time must be explicitly incorporated into the analytical framework. Exposures should be modelled across multiple temporal scales—acute, subacute, and chronic—using approaches such as lagged variables, moving averages, cumulative exposure indices, and life-course or developmental windows [32,34,35,36,37,40]. This distinction is critical, as the biological effects of short-term pollutant spikes may differ substantially from those of sustained exposure, and early-life environmental influences may not directly translate into adult disease activity. Capturing temporality enables the model to reflect the dynamic and evolving nature of AD.

### 5.3. Susceptibility/Modifier Layer

Host-specific factors shape individual variability in response to environmental exposures. These include genetic susceptibility (including epidermal barrier function and filaggrin status), demographic characteristics (age, sex, ethnicity), baseline disease severity, allergic sensitization, microbiome composition, treatment intensity or adherence, and psychological burden [5,29,48,56,57,58,59,60]. Treatment intensity and adherence should be recorded separately as potentially time-varying modifiers rather than intrinsic susceptibility traits. Incorporating these modifiers may help characterize differences in exposure–response relationships and inform individualized risk prediction.

### 5.4. AI Integration Layer

This analytical core integrates and processes data across all layers. It encompasses feature engineering, multimodal data fusion, and the application of supervised learning models to predict disease severity and flares.

Model selection should reflect the intended task, data structure, and available sample size, with longitudinal approaches accounting for temporal dependencies.

Unsupervised approaches may identify patient clusters, which should not be interpreted as biological endotypes without evidence of reproducibility and distinct underlying biological mechanisms. Explainability modules can clarify how features contribute to model predictions, but do not establish causal relationships or explain disease mechanisms [55,61].

Where feasible, causal inference techniques may be incorporated to investigate explicitly defined exposure or treatment effects under appropriate study designs and assumptions, accounting for time-varying treatment-related confounding when relevant [27,49,50]. The goal is to generate robust, interpretable predictions and testable hypotheses, with clinical utility requiring separate evaluation.

### 5.5. Output Layer

The framework is intended to support a range of clinically and scientifically relevant outputs. These include real-time severity estimation, short-term flare prediction, classification of longitudinal disease trajectories, identification of exposure-responsive patient subgroups, prediction of treatment response, and prioritization of candidate biomarkers. These outputs differ in their evidential basis: selected severity-assessment and longitudinal prediction tasks have been evaluated in AD, whereas exposome-informed treatment selection and personalized prevention remain proposed applications. For clinicians, potential outputs include interpretable risk scores, individualized trigger profiles, and alerts for high-risk periods. For research, the framework could contribute to refined phenomapping and the generation of testable biological hypotheses.

### 5.6. Learning Health Loop

Finally, the framework is inherently iterative. Data collected during routine clinical care or longitudinal observational studies can be continuously reintegrated to update and recalibrate models, with any improvement in performance and generalizability requiring subsequent evaluation. This adaptive learning loop is particularly important in AD, where new therapeutic options are reshaping disease trajectories and risk profiles. Changes in patient populations, treatment practices, or measurement methods may produce dataset shift and deterioration in predictive performance over time. Model updating should therefore follow a predefined governance process, with documented versions, ongoing performance monitoring, and revalidation before updated models are introduced into clinical practice [62].

## 6. Potential Clinical and Research Applications

An exposome–AI framework in AD could support several closely connected applications rather than a set of isolated functions. Applications already investigated in AD include severity assessment and short-term severity prediction. By integrating recent exposures with baseline susceptibility, treatment context, prior disease dynamics, and digitally captured behavioural signals, such models could potentially improve estimates of current and near-future disease activity, although their added value over clinical information and previous symptom trajectories requires evaluation [26,27,49,50,51,56,57,60,63]. This is particularly relevant in AD, where disease expression is inherently dynamic. Indeed, clinical severity scores, patient-reported symptoms, and wearable-derived scratching measures capture different dimensions of disease burden and should not be treated as interchangeable outcomes [64,65,66].

Predicting exacerbations represents a clinically relevant but still evolving objective. The ability to anticipate disease worsening could lead to earlier interventions, therapeutic adjustments, and better exposure prevention. These potential benefits remain to be demonstrated for an integrated exposome–AI system. At present, however, this perspective should be considered with due caution, because, although environmental modeling in atopic dermatitis is clearly feasible, the incremental predictive contribution of environmental variables beyond previous disease activity remains uncertain [53,67,68]. Moreover, studies investigating predictors of annual flare burden should not be interpreted as validation of short-term alerts for imminent exacerbations.

The clinical relevance of these applications depends on clearly defined outcomes and prediction horizons. Clinical signs, patient-reported symptoms, and itch intensity should be assessed using appropriate instruments, such as EASI, POEM, and a peak 24-h itch numerical rating scale, respectively [69]. Flare prediction also requires an explicit operational definition; worsening requiring treatment escalation is one studied approach, provided that escalation criteria are agreed at baseline [70]. Prediction horizons, such as 24 h or 7 days, should be selected according to the intended clinical use and supported by an appropriate measurement schedule. For future studies, a possible schedule could include daily symptom and treatment records, weekly POEM assessments, and EASI measurements at prespecified clinical visits. These prediction horizons and monitoring schedules are illustrative examples for future studies and should not be interpreted as validated recommendations.

Similarly, treatment-response prediction requires a specified therapy, response criterion, and assessment time point. An alert indicating increased risk within the selected horizon could prompt review of symptoms, adherence, and the management plan. Its clinical value would depend on whether this approach improves outcomes compared with usual care, while limiting unnecessary interventions and false alerts.

Beyond prediction, the integration of exposomic and AI-based approaches may be especially valuable for characterizing disease heterogeneity. Rather than remaining confined to conventional clinical labels, unsupervised and integrative modelling may delineate patient subgroups with distinct exposure and biological profiles defined by convergent patterns of barrier dysfunction, immune polarization, microbial imbalance, sensitization profile, and molecular signatures [33,34,36,37,39,40,43,58]. In this setting, exposome data may reveal clinically relevant heterogeneity that morphology alone cannot adequately capture and may help link observable phenotypes to underlying molecular endotypes [61,71]. However, a data-driven cluster should not be considered a biological endotype without reproducibility and evidence of distinct underlying mechanisms.

The same conceptual framework could also be useful for identifying potential biomarkers. For example, linking longitudinal outcomes with specific profiles (e.g., microbiome, barrier lipids, inflammatory mediators, and broader multi-omics levels) may identify potential biomarkers not only of disease severity, but also exposure-related responses, propensity to relapse, or imminent transition to the acute phase [32,33,34,42,43,48,58,59]. From this perspective, these biomarkers are no longer static and could instead assume the role of dynamic indicators. However, most of these biomarkers still require prospective and external validation before routine clinical use [71,72].

Ultimately, these potential applications converge toward the broader goal of treatment personalization. Exposome-aware models could help identify patients who may benefit from intensified barrier-directed care, targeted intervention for sleep disturbance and scratching, environmental counselling, or closer follow-up despite apparently mild baseline severity. More broadly, they could support a precision framework in which treatment decisions are informed not only by visible inflammation, but also by molecular endotype, exposure sensitivity, and real-world disease behaviour [8,39,73]. Within the proposed integrated framework, these remain research goals rather than validated treatment-selection strategies. For this framework to have clinical utility, predictive accuracy alone will not be enough. Its outputs must also be interpretable, robust, externally validated, and developed on representative datasets that limit bias. Predictions that cannot be anchored to clinically meaningful determinants are unlikely to guide decision-making effectively. In AD, these features should be viewed not as optional refinements, but as core prerequisites for clinical translation (Table 1) [51,59,66].

## 7. Methodological Priorities and Sources of Bias

Several methodological conditions must be satisfied for an AI-enabled exposome framework in AD to achieve scientific credibility and clinical relevance (Supplementary Table S2).

First, exposure assessment requires substantial improvement. While area-level proxies—such as regional air pollution or meteorological data—are widely used, they provide only an indirect approximation of individual exposure. Future studies should move toward integrating these estimates with patient-level data, including wearable sensor measurements, indoor environmental monitoring, behavioural logging, and repeated biological sampling [74,75,76]. This multimodal approach could support a more precise and personalised characterisation of the exposome, capturing both external and internal exposure dynamics.

Treatment-related confounding must be addressed as a central methodological challenge rather than a secondary concern. In AD, therapeutic interventions are inherently dynamic: they are initiated or intensified in response to worsening disease, while simultaneously modifying disease activity. This bidirectional relationship creates a high risk of bias in naïve predictive or associative models. For prediction, medication use, treatment intensity, and adherence should be considered when available at the time of prediction. Their inclusion as predictors does not, however, resolve causal confounding. Analyses estimating exposure or treatment effects require methods appropriate to the temporal relationship between disease activity and treatment.

Also, missing data and temporal misalignment represent pervasive issues in exposome research. Data streams derived from environmental monitoring, patient-reported outcomes, and biological sampling are rarely synchronized and often incomplete. Robust preprocessing pipelines are therefore essential and should include methods for handling irregular time intervals, imputing missing values, constructing biologically meaningful lag variables, and addressing multicollinearity among correlated exposures. Without such harmonisation, downstream analyses risk instability or misinterpretation. Model complexity should be matched to the available sample size to reduce the risk of overfitting. Including more predictors does not necessarily improve accuracy; the added predictive value of exposure and biological variables should be evaluated against a reference model based on clinical information and previous disease activity.

Data-dependent preprocessing, feature selection, and hyperparameter tuning should be confined to the training data within each validation split. Validation should reflect the intended use of the model. When assessing generalizability to new patients, all observations from a given individual should remain within either the development or evaluation dataset. For longitudinal forecasting, temporal order should also be preserved, including when predicting future outcomes in patients already monitored [77].

Moreover, fairness and generalizability are critical considerations. Model performance and exposure patterns may vary substantially across populations defined by skin tone, age, socioeconomic status, and geographic context. Failure to account for this diversity may lead to biased predictions and reduced clinical utility in underrepresented groups. Accordingly, training datasets should be diverse, and models must undergo rigorous external validation across multiple, heterogeneous cohorts to assess robustness and equity [78,79]. Where sample sizes permit, performance should also be evaluated across relevant demographic subgroups.

Interpretability should be embedded within the modelling process. For AI tools to be adopted in clinical practice, they must provide transparent, explainable outputs that clinicians can understand and trust. This includes clear reporting of feature importance, model calibration, and demonstration of added clinical value over existing approaches. Finally, model validation should extend beyond conventional accuracy metrics. Internal validation estimates optimism in model performance within the development dataset. Temporal validation evaluates performance in subsequently collected data not used for model development, whereas geographical external validation assesses performance in other centres or regions. Calibration describes agreement between predicted and observed outcomes, while clinical utility concerns whether predictions usefully inform clinical decisions. Prospective comparative studies are needed to determine whether model-guided care improves patient outcomes [80]. A comprehensive evaluation should include discrimination, calibration, clinical usefulness (e.g., decision curve analysis), transportability across settings, and robustness to real-world data limitations, such as missingness. In the context of the exposome, biological plausibility is also essential: model-derived associations should be assessed against existing biological knowledge and regarded as hypotheses requiring further investigation. Even highly predictive models are unlikely to influence clinical care if they lack interpretability, reproducibility, or external validity.

## 8. Ethical, Regulatory, and Implementation Considerations

The proposed exposome–AI framework also poses significant ethical and implementation challenges that must be addressed to ensure responsible and equitable use. Continuous monitoring of environmental and behavioural data may raise significant privacy concerns, precisely because it uses geolocation data, home sensors, and wearable devices. These data streams may reveal sensitive information about daily routines, living conditions, and personal habits. In parallel, digital inequity remains a major risk: populations with limited access to technology or lower digital literacy may be underrepresented in datasets, leading to biased models and potentially widening existing health disparities. Additionally, both patients and clinicians may be reluctant to adopt systems perceived as opaque, burdensome, or overly deterministic in their recommendations.

These concerns highlight the importance of human-centered design. AI systems should therefore be developed to support clinicians and researchers, not to replace clinical judgment. This concept is crucial to ensuring transparent and collaborative decision-making. For these reasons, a phased implementation strategy would allow clinical applications to be evaluated progressively. For example, initial applications could focus on research-oriented dashboards that integrate symptom trajectories with environmental exposures and interpretable risk estimates. Over time, these tools could evolve into clinical decision support systems. Even further down the line, these tools could be made available to patients through apps that provide personalized information. Throughout this process, adherence to established reporting standards and robust validation frameworks will be necessary.

## 9. Research Agenda

A realistic research agenda can be developed in stages. The first step is to build consensus by defining a core dataset regarding the AD exposome, outlining preferred outcomes, establishing minimal metadata, and recommending appropriate temporal granularity.

The second step involves designing a prospective cohort study that integrates clinical phenotyping, geospatial exposure linking, digital symptom capture, and at least one biological layer, such as microbiome or transcriptomics. Analyses should assess whether these additional data improve prediction beyond clinical information and previous disease activity.

The third step focuses on external validation across different climates, healthcare systems, and patient subgroups.

The fourth step consists of pragmatic testing to determine whether exposome-informed risk models can improve outcomes, such as flare frequency, sleep quality, rescue treatment usage, or overall quality of life.

The field should avoid the temptation to jump straight to app-based prediction without solid biological and methodological foundations. The potential value of the exposome–AI framework lies not only in predicting severity but also in investigating how cumulative environmental burdens interact with individual susceptibility over time. In this way, AD could serve as a model disease for precision environmental dermatology.

## 10. Conclusions

Integrating the exposome and AI offers a conceptual framework for examining how environmental exposures, host susceptibility, and biological responses shape the course of AD over time. By bringing these dimensions into a common analytical perspective, the proposed framework could help characterize disease heterogeneity and generate testable hypotheses about exposure–response relationships.

Current evidence supports selected AI applications in AD, including image-based severity assessment, longitudinal severity prediction, and automated scratch monitoring. These advances provide the basis for further investigation, but do not establish the clinical utility of an integrated exposome–AI system. Exposure-responsive stratification, biomarker discovery, and individualized prevention remain research objectives. A central question is whether incorporating exposure data improves prediction beyond clinical information and previous disease activity, and whether any improvement translates into better patient care.

Addressing these questions will require prospective longitudinal studies with clearly defined outcomes, reliable exposure measurements, and appropriate consideration of treatment-related confounding. Evaluation should encompass calibration, interpretability, and fairness, with external validation across diverse populations and settings. Comparative studies will then be needed to determine whether model-guided management offers meaningful benefit over usual care. The contribution of this framework to precision dermatology will ultimately depend on its ability to connect environmental complexity with reproducible findings and clinically useful decisions.

## Figures and Tables

**Table 1:** Evidence status of selected AI applications in AD and proposed applications within the integrated exposome–AI framework.

Evidence Status	Applications	Interpretation
Evaluated in AD studies	Image-based assessment of clinical signs; longitudinal severity prediction; automated scratch detection and quantification	Specific tasks have been evaluated in AD datasets. This does not establish the clinical utility of a complete exposome–AI system.
Exploratory	Environmental severity modelling; prediction of annual flare burden; transcriptome–microbiota classification and candidate biomarker identification	Selected data combinations and predictive relationships have been investigated. Their generalizability and clinical value require further evaluation.
Proposed objectives	Exposome-informed short-term flare alerts; exposure-responsive patient stratification; treatment-response prediction; personalized prevention	Intended applications of the proposed framework requiring prospective validation and assessment of clinical benefit.

## Data Availability

No new data were created or analyzed in this study. Data sharing is not applicable to this article.

## Supplementary Materials

The following supporting information can be downloaded at: https://www.mdpi.com/article/10.3390/ijms27209209/s1.
