# Supplementary material for: Atopic Dermatitis in the Era of Exposome and Artificial Intelligence
Source: Int J Mol Sci. 2026 Oct 16;27(20):9209. doi: 10.3390/ijms27209209 (PMC13705081; doi:10.3390/ijms27209209)
Supplement: Supplementary file 1 [file ijms-27-09209-s001.zip › ijms-4608992-supplementary.pdf]

**Supplementary materials**

**Table S1**. Core domains of the proposed AD exposome-AI framework.

| Domain | Examples | Potential AI/clinical relevance |
|---|---|---|
| External general exposome | Air pollution, weather, climate, green space, traffic, housing, tobacco smoke | Short-term flare risk, severity trends, contextual risk stratification |
| External specific exposome | Allergens, detergents, skin care, diet, infections, stress, sleep, behavior | Patient-level trigger profiling, personalized prevention |
| Internal exposome | Barrier lipids, filaggrin-related biology, cytokines, microbiome, omics signatures | Endotyping, biomarker discovery, mechanism-informed prediction |
| Treatment layer | Topical/systemic therapy, adherence, escalation, rescue medication | Confounding control, response modelling, dynamic prognosis |
| Temporal layer | Lag effects, moving averages, seasonality, life-course windows | Flare forecasting, trajectory analysis, cumulative burden estimates |
| AI integration layer | Prediction, clustering, explainability, multimodal fusion, causal modelling | Clinically actionable risk scores and exposome-sensitive phenotypes |

**Table S2.** Methodological priorities for exposome-AI studies in AD.

| Priority | Why it matters | Suggested solution |
|---|---|---|
| Exposure assessment | Area-level estimates may misclassify individual exposure | Combine geospatial linkage with patient-level sensing and diaries |
| Temporality | AD is dynamic; exposure effects vary by lag and duration | Use repeated measures, lagged features, moving averages and trajectory models |
| Treatment-confounding | Therapy both reflects and changes disease activity | Model treatment intensity and adherence explicitly |
| Missingness/alignment | Real-world multimodal data are asynchronous and incomplete | Use harmonization pipelines, principled imputation and timestamp governance |
| Interpretability | Black-box predictions limit trust and actionability | Use explainable AI, calibration analysis and clinically meaningful outputs |
| Generalizability/fairness | Performance may vary by geography, age, ethnicity and skin tone | Develop diverse cohorts and require external validation |
